# Improving RNA-Seq expression estimation by modeling isoform- and exon-specific read sequencing rate

**DOI:** 10.1186/s12859-015-0750-6

**Published:** 2015-10-16

**Authors:** Xuejun Liu, Xinxin Shi, Chunlin Chen, Li Zhang

**Affiliations:** College of Computer Science and Technology, Nanjing University of Aeronautics and Astronautics, 29 Jiangjun Rd., Nanjing, 211106 China

**Keywords:** Transcript expression, Gene expression, RNA-Seq data analysis, Latent dirichlet allocation, Probabilistic model

## Abstract

**Background:**

The high-throughput sequencing technology, RNA-Seq, has been widely used to quantify gene and isoform expression in the study of transcriptome in recent years. Accurate expression measurement from the millions or billions of short generated reads is obstructed by difficulties. One is ambiguous mapping of reads to reference transcriptome caused by alternative splicing. This increases the uncertainty in estimating isoform expression. The other is non-uniformity of read distribution along the reference transcriptome due to positional, sequencing, mappability and other undiscovered sources of biases. This violates the uniform assumption of read distribution for many expression calculation approaches, such as the direct RPKM calculation and Poisson-based models. Many methods have been proposed to address these difficulties. Some approaches employ latent variable models to discover the underlying pattern of read sequencing. However, most of these methods make bias correction based on surrounding sequence contents and share the bias models by all genes. They therefore cannot estimate gene- and isoform-specific biases as revealed by recent studies.

**Results:**

We propose a latent variable model, NLDMseq, to estimate gene and isoform expression. Our method adopts latent variables to model the unknown isoforms, from which reads originate, and the underlying percentage of multiple spliced variants. The isoform- and exon-specific read sequencing biases are modeled to account for the non-uniformity of read distribution, and are identified by utilizing the replicate information of multiple lanes of a single library run. We employ simulation and real data to verify the performance of our method in terms of accuracy in the calculation of gene and isoform expression. Results show that NLDMseq obtains competitive gene and isoform expression compared to popular alternatives. Finally, the proposed method is applied to the detection of differential expression (DE) to show its usefulness in the downstream analysis.

**Conclusions:**

The proposed NLDMseq method provides an approach to accurately estimate gene and isoform expression from RNA-Seq data by modeling the isoform- and exon-specific read sequencing biases. It makes use of a latent variable model to discover the hidden pattern of read sequencing. We have shown that it works well in both simulations and real datasets, and has competitive performance compared to popular methods. The method has been implemented as a freely available software which can be found at https://github.com/PUGEA/NLDMseq.

**Electronic supplementary material:**

The online version of this article (doi:10.1186/s12859-015-0750-6) contains supplementary material, which is available to authorized users.

## Background

RNA-Seq, based on high-throughput sequencing technology, is nowadays a commonly used method to study transcriptome. An RNA-Seq experiment typically produces millions or billions of short sequenced reads. By counting the reads aligned to a reference transcriptome, the expression of the related transcripts can be calculated [1]. As alternative splicing, where various protein isoforms are yielded due to the different exon constitutions for a gene, receives more and more interest in the research of biomedicine, RNA-Seq has been widely used to quantify gene and isoform expression.

As read counts are proportional to the mRNA fragment abundance of a gene, the reads per kilobase of transcript per million mapped reads (RPKM) has been commonly used to represent gene expression [2]. Due to the existence of alternative splicing, multiple isoforms can share exons of a gene (we use isoforms to represent splice variants hereafter for simplicity). Therefore, there are a large number of reads which cannot be uniquely aligned to the isoform they originate from. For this reason, reads mapped to the shared exon need to be deconvoluted before computing isoform expression. Inferring the mechanism of dispatching mapped reads to their origins is a difficulty in isoform expression calculation from RNA-Seq data. A number of approaches make use of the additive property of Poisson distribution to address this problem [3–5]. Given a transcriptome assembly, the sequencing rate for a read count can be factorized as a sum of contributions from all isoforms to which this read can be mapped. Another group of methods employ latent variable models to discover the unknown isoform expression [6–8]. By introducing discrete latent variables to representing isoforms each read originates from, the sequencing process of each individual read is modeled.

In addition to read mapping ambiguity, another difficulty of expression calculation from RNA-Seq data is the non-uniformity of read distribution along the reference genome and transcriptome due to positional, sequencing, mappability and other undiscovered sources of biases [9]. The non-uniform read distribution violates the assumption of the direct RPKM representation and many expression calculation approaches, such as Poisson-based models. In order to obtain accurate expression estimates, these biases have to be accounted in expression calculation. Consequently, many bias correction strategies have been proposed to relieve the influence of non-uniformity of read distribution [5, 9–13]. Most of these approaches estimates the overall biases based on surrounding sequence contents or empirical data, and bias models are mostly shared by all genes. For example, the commonly used method, Cufflinks [12, 14], used a variable length Markov model to learn sequence-specific and positional biases based on the surrounding sequences of all reads in a set of empirical data. This model was then shared for all genes to make bias correction. Therefore, if two reads have similar nucleotide constitution and similar relative positions along transcriptome reference, they would have the similar biases under this model assumption. A recent study found that the read distribution is isoform-specific and a Poisson-based model, Sequgio, is proposed to jointly estimate isoform expression and isoform-specific read sequencing bias [15].

In this paper, we propose a latent variable model, NLDMseq (normalized latent Dirichlet-Multinomial model for RNA-Seq data), to estimate gene and isoform expression. Given known annotations, this model introduces latent variables to represent the unknown isoforms, from which reads originate, and the proportion of multiple spliced isoforms. The isoform- and exon-specific read sequencing rates are modeled as parameters which can be estimated from data. We utilize the replicate information of multiple lanes for a single library to identify read sequencing rates. NLDMseq benefits from the latent variable model that would help to discover the hidden pattern of read sequencing. Another advantage of NLDMseq is that it models normalized read counts for each exon rather than each individual read as many latent variable models do [6–8]. Therefore, NLDMseq does not need to deal with the nucleotide constitution of each read, and thus achieves fast computation. We employ simulation and real data to verify the performance of our method in terms of accuracy in the calculation of gene and isoform expression.

## Methods

### Latent dirichlet-multinomial model

It has been found by previous studies that there exist similarities among the patterns of count variation for many genes or isoforms among samples [10, 15]. There is therefore a need to model read distribution bias for each individual gene or isoform. To reach this goal, it would be ideal to model the sequencing rate for each individual read of a particular gene or isoform. However, this would bring tremendous computation in light of the huge amount of read data. Also, for many lowly expressed genes overfitting would be a problem with little data available. Considering computational efficiency of the model, we count read number for each exon in the transcriptome reference and take exon as the basic unit of data to model exon-specific read sequencing rate for each isoform. As long reads and paired-end reads become more and more popular in RNA-seq protocal, we use fragment to represent any transcript sequence found in sample and are then interested in fragment number for each exon hereafter. We aim to model the stochastic mechanism in generating the “exon corpora” of RNA-seq data for each gene.

**Fig. 1:**
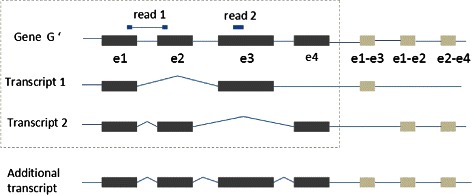
An example of gene structure with junction exons and the additional noise isoform. This example gene originally contains four exons, e1, e2, e3 and e4, and two annotated splice variants, isoform1 and isoform2, as shown in the dotted rectangle. According to the constitution of the two splice variants, three junction exons are constructed, e1-e3, e1-e2 and e2-e4. We also include an additional splice variant containing all annotated and junction exons as shown on the bottom of the figure. On the top of the figure, we show two example reads, read1 and read2, among which read1 was aligned to the junction of e1 and e2, and read2 was aligned to e3. When counting the fragment number for each exon, we add one to the fragment counts of e1-e2 and e3 respectively

Given a known annotation, assume there are *E* exons and *K* isoforms for a given gene. Let *n*_*i*_ represent the number of fragments that fall in the *i*th exon, where 1≤*i*≤*E*. We also consider the exon junctions and treat them as individual exons in order to obtain more information of alternative splicing in the data. Let *L* represent the length of reads. We construct special junction exons by combining the sequence with length *L*−1 at the end of the exon ahead and the sequence with the same length at forepart of the next exon (see the example in Fig. 1). For paired-end data, we count the fragment between a pair of reads. If the fragment covers multiple exons, we consider the average length of fragments and add one to the fragment count of every exon allowed by it. Overlapping exons are divided into multiple exons to avoid the redundant fragment counting. To remove the bias of the length of exons in fragment counts, *n*_*i*_ is normalized according to the exon length. In the following design of NLDMseq, we take *n*_*i*_ as a quantity measuring the frequency of observing the *i*th exon in experimental data.

We denote *e* as an indicator vector with *E* entries, among which there is only a single entry with value 1 and all the others are zero. Thus, each vector represents an exon. For each observed exon *e*, we assume there is an associated latent variable *t* indicating the unknown isoform which generates this exon. The *K*-vector *t* has value one for the *k*th entry if the exon is generated by isoform *k*, and 0 for others. For the purpose of accommodating the random noise in fragment sequencing and fragments generated from undiscovered isoforms, we introduce an additional isoform which contains all exons of this gene. If the possibility of the generation for a particular exon from all known isoforms is considerably low, it may be assigned to this special isoform automatically. Figure 1 shows the construction of gene structure and fragment counting strategy of an example gene. A *K*-vector variable *θ* is used to denote the percentage of the *K* isoforms, where *θ*_*i*_>0 and $\sum _{i}\theta _{i}=1$. We assume a Dirichlet prior on *θ*.

With the above assumptions and notations, we adopt the Dirichlet-Multinomial Bayesian model as that in the latent Dirichlet allocation [16]. The data we consider here are a single library run on multiple lanes. The dataset for each gene is the normalized fragment counts {*n*_*i*_} for all included exons. We denote *N* as the total number of the observed exons, where $N=\sum _{i} n_{i}$. The generative process of data {*e*_*n*_} (1≤*n*≤*N*) for each gene is assumed as follows,

1. Generate *θ*_*l*_ once for lane *l*, *θ*_*l*_∼Dirichlet(*α*).
2. For lane *l*, generate *t*_*ln*_, *t*_*ln*_∼Multinomial(1,*θ*_*l*_).
3. Generate exon *e*_*ln*_ given *t*_*ln*_, $e_{\textit {ln}}\sim \text {Multinomial}(1,\beta ^{t_{\textit {ln}}})\phantom {\dot {i}\!}$.

**Fig. 2:**
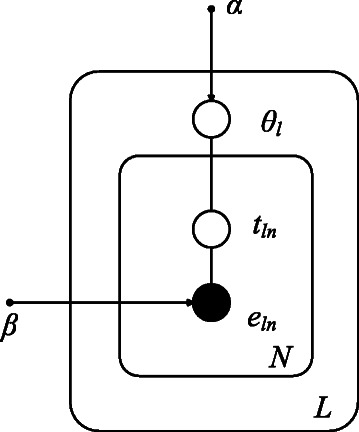
Graphic model representation of NLDMseq. The while circles represent latent variables, the large black circle for the observed exon and the small balck circles for hyperparameters. The plates denote replication of the random variables

By repeating steps 2 and 3, all exon data can be generated for each lane. In our model, *e* is the observed data, *θ* and *t* are latent variables, and the *K*-vector *α* and the *K*×*E* matrix *β* are hyperparameters, where *α*_*k*_>0, ${\beta _{j}^{t}}>0$ and $\sum _{j}{\beta _{j}^{t}}=1$. The vector *β* ^*t*^ is the *k*th row of *β* corresponding to isoform *t*. We can see that under our model assumption the parameter *β* ^*t*^ is isoform-specific and each entry indicates the average sequencing rate for an exon. For those exons which are not used in isoform *t*, the corresponding entries in *β* ^*t*^ are constrained to zero. Here, we consider to model the data for each individual lane in order to make use of this technical replicate information to have better estimation of the distribution of *θ* and the isoform- and exon-specific read sequencing rate *β*. A graphical representation of our model is shown in Fig. 2 where the relations between all these variables can be found.

We notice that Sequgio proposed in [15] also modeled the isoform-specific read sequencing bias. Our model differs from Sequgio in the fact that Sequgio uses the simple Poisson distribution to model the generated reads and does not model the variability in the selection of isoform which generates each read. Also, we do not share the isoform-specific read sequencing rate *β* across samples as Sequgio in case that biological variation may violate the conserved read count distribution for some genes. Biological samples are therefore processed individually with our approach. We aim to provide accurate expression measurements at this stage and intend to handle the biological replicate noise in the downstream analysis (see the section of “Application to DE analysis”). NLDMseq is also distinct from other latent variable models, such as RSEM [6], MISO [7] and Bitseq [8], which explicitly modeled the generation process of each read by examining the the start sequencing position and nucleotide components of each read. These approaches either assume a uniform read distribution along isoform sequence [7] or need to deal with nucleotide constitution in order to correct the bias in read distribution [6, 8]. Consequently, this would increase the computation cost of models. In contrast, our method models the frequency of the existence of each exon and this is able to reduce the computation load of our model. Meanwhile, the isoform- and exon-specific read sequencing rate is also obtained and this accounts for the non-uniformity of the primary read distribution.

### Variational EM solution

Our purpose is to infer the hidden generative process which most likely produces the observed normalized exon counts {*n*_*li*_}. In this process we are interested in the posterior of *θ*, *P*(*θ*|{*e*_*ln*_}) which indicates the underlying fraction of isoform abundances. Unfortunately, this distribution is intractable to compute when we apply Bayes’ rule. We employ the variational EM algorithm proposed in [16] to work out NLDMseq. Assuming the independence of {*θ*_*l*_} and {*t*_*ln*_}, the posterior of {*θ*_*l*_} and {*t*_*ln*_} can be approximated by a set of Dirichlet distributions with parameters {*η*_*l*_} and a set of multinomial distributions with parameters {*λ*_*ln*_}, respectively. Initially, we set unbiased values for the hyperparameters *α* and *β*, indicating that no preference is set for any isoform and exon. At the E-step, the variational parameters are updated as follows,

(1) $$\begin{array}{*{20}l} \lambda_{lnk}&=\beta_{kj}\exp\left\{\left<\log\left(\theta_{lk}\right)\right>_{q(\theta_{l})}\right\} \end{array} $$

(2) $$\begin{array}{*{20}l} \eta_{lk}&=\alpha_{k}+\sum\limits_{ln}\lambda_{lnk},  \end{array} $$

where < · > represents expectation and *q* denotes the variational distribution of *θ*_*l*_. We omit the variational parameters of *q* distribution for simplification. The Eqs. (1) and (2) are updated iteratively until convergence is obtained.

At the M-step, the following function is maximized with respect to *α* and *β*,

(3) $$ \mathcal{L}\left(\alpha,\beta\right)=\left<\log p\left(\{\theta_{l}\},\{t_{ln}\},\{e_{ln}\}|\alpha,\beta\right)\right>_{q\left(\{\theta_{l}\}\right)q\left(\{t_{nl}\}\right)},  $$

where

(4) $$ \begin{aligned} &p\left(\{\theta_{l}\},\{t_{ln}\},\{e_{ln}\}|\alpha,\beta\right)\\ &\qquad=\prod\limits_{l} p\left(\theta_{l}|\alpha\right)\prod\limits_{n} p\left(t_{ln}|\theta_{l}\right) p\left(e_{ln}|t_{ln},\beta^{t_{ln}}\right). \end{aligned}  $$

The hyperparameter *β* can be worked out in a closed form as follows,

(5) $$ \beta_{kj}=\sum\limits_{l}\sum\limits_{n}\lambda_{lnk}e_{lnj}.  $$

If isoform *k* includes exon *j*, *β*_*kj*_ is updated using Eq. (5), otherwise it is confined to be zero. The hyperparameter *α* cannot be solved analytically and therefore can be worked using the efficient Newton-Raphson method which was used in [16]. The E-step and M-step are repeated until a stable solution is achieved. Readers can refer to [16] for details of the variational EM algorithm.

### Gene and isoform expression representation

Since *p*(*θ*_*l*_|{*e*_*ln*_}) implies the percentage of multiple isoform abundance for each lane, we use the generative distribution *p*(*θ*|*α*) with the estimated *α* to represent the underlying fraction of isoform abundance. The expectation of *θ*_*k*_ is

(6) $$ \left<\theta_{k}\right>=\frac{\alpha_{k}}{\sum_{k}\alpha_{k}},~\text{and}~\sum\limits_{k}\left<\theta_{k}\right>=1.  $$

The fragments mapped to each exon are allocated to the corresponding isoforms according to the fraction in Eq. (6). Using the FPKM representation in [14], the expression of the *k*th isoform, *f*_*k*_, can be expressed as

(7) $$ f_{k}=\frac{10^{9}\theta_{k}}{N_{s}}\sum\limits_{li}n_{li},  $$

where *N*_*s*_ is the total number of fragments obtained for this sample. Gene expression can be represented by $\sum _{k}f_{k}$. In order to obtain a level of measurement uncertainty for both isoform and gene expression, we draw *M* samples from the distribution *p*(*θ*|*α*) and calculate isoform and gene expression for each sample *θ* ^*m*^, where 1≤*m*≤*M*. Thus, we have *M* estimates for each isoform and gene expression. Using these samples, we can obtain the variance of the estimated expression which can be propagated into downstream analysis to obtain improved results. For single-isoform genes, the expression is calculated directly using FPKM assuming the uniform distribution of the reads.

### Software

We have implemented NLDMseq in a free Python/C tool, which is available from https://github.com/PUGEA/NLDMseq, for public use. Any aligner that can align the raw experimental reads to reference transcriptome can be used for the preprocess of RNA-seq data for NLDMseq. We use Bowtie 2 [17] throughout this paper since it is sensitive to gaps and good at aligning long reads, and is used by many approaches, such as Bitseq [8] and MMSEQ [4]. After the alignment, our software can be used to calculate gene and isoform expression. Our software includes two parts. One is Python scripts which are used to expand gene models and obtain the fragment count for each exon from the alignment output file. The other is C codes, which solve the NLDMseq model and compute expression measurements. For simplicity of usage, the software can be called in a single run. For details of using our software, readers can refer to the documentation and examples on the software’s website.

### Datasets

We use the well-studied Microarray Quality Control (MAQC) dataset [18] to validate gene expression estimation from NLDMseq. Gene expression from high-quality RNA samples is measured in MAQC project to assess the comparability across multiple platforms mainly including various microarray and next-generation sequencing technologies. Two RNA samples, the universal human reference (UHR) RNA and the human brain reference (HBR) RNA, from Illumina platform are selected in our work to verify our new method, including single-end data (SRA010153) and paired-end data (SRA012427). The SRA010153 data contains two samples, HBR and UHR, while the SRA012427 data contains a single sample UHR. Besides genes in the whole human genome measured by microarray and RNA-Seq, around one thousand genes have been measured by qRT-PCR experiments. These genes can be used as gold standard to validate gene expression estimation obtained from other platforms. This dataset has been widely used as the benchmark to verify various analysis methods for microarray and RNA-Seq technologies [19–21]. Among the qRT-PCR validated genes, we used the Ensembl annotation data (GRCh37/hg19) and obtained 740 matching multi-isoform genes (see Additional file 5: Table S1). These genes are used to evaluate the performance of our approach at gene level. We further use the method in [20] to filter 198 differential expression (DE) genes and 81 non-DE multi-isoform genes (see Additional file 2: Table S2) with high confidence according to qRT-PCR measurements. Data of these 279 qRT-PCR validated genes is used as a gold standard to evaluate the sensitivity and the specificity of our method applied to DE analysis.

A real study on molecular injury in response to tobacco smoke exposure and lung cancer pathogenesis (SELC) [22] is used to further evaluate the performance of our approach on gene expression calculation. This experiment involves four phenotypes, healthy never smokers (NS), current smokers (S), smokers with (C) and without (NC) lung cancer undergoing lung nodule resection surgery. RNA-seq libraries were prepared and sequenced to obtain 22 million 75 nt paired-end reads for each sample by using the standard Illumina mRNA-seq protocol. The study considered differential gene expression in the comparisons, S vs. NS and C vs. NC. The RNA-seq measurements of eight genes were validated using qRT-PCR data. We aligned the reads to Ensembl annotation (GRCh37/hg19) and found one of the eight validated genes, NFKB1A1, not be annotated. We therefore exclude this gene and use the obtained fold changes of the other seven genes in expression comparison.

We also use a publicly available human breast cancer (HBC) dataset [23] to verify the performance of NLDMseq on isoform expression estimation. This dataset includes two samples, human breast cancer cell line (MCF-7) and normal cell line (HME). Four multi-isoform genes (TRAP1, ZNF581, WISP2 and HIST1H2BD) were validated by qRT-PCR experiments [24]. Each gene has two isoforms which have been interrogated for both cell lines. According the experiment results in [24], the regulations of these eight isoforms in the two samples and the regulations of the two isoforms for the same gene in the same sample can be calculated. We therefore obtain 16 qRT-PCR validated regulations which can be used to verify the performance of various approaches on isoform expression calculation (see Additional file 7: Table S3).

We generated simulated data using our model based on the calculated parameters from the MAQC dataset. We select all 1,604 multi-isoform genes and 12,064 isoforms of chromosome 1 from Ensembl human genome assembly GRCh37/hg19 and simulate two samples with single-end and paired-end data respectively. For the single-end data, we generate 3,673,804 single-end reads with seven lanes using parameters $\hat {\beta }$ calculated from the single-end data for HBR sample. For the paired-end data we generate 125,126 reads with three lanes using parameters $\hat {\beta }$ calculated from the paired-end data for UHR sample. For both cases, the values of parameters $\hat {\alpha }$ are randomly generated. For each simulated read, we first sample *θ*_*l*_ from $\text {Dirichlet}(\hat {\alpha })$. Second, for each lane generate *t*_*ln*_ from Multinomial(1,*θ*_*l*_). Next, given the generated *t*_*ln*_ generate exon *e*_*ln*_ from $\text {Multinomial}(1,\beta ^{t_{\textit {ln}}})\phantom {\dot {i}\!}$. Finally, uniformly sample the starting position along exon *e*_*ln*_ for a single-end read. To generate a pair of paired-end reads, we sample the length of the sequenced fragment from N(206,19.6) and then uniformly sample the starting position of this fragment along exon *e*_*ln*_. With the sampled fragment position and length, the paired reads for both ends can be simulated.

## Results and discussion

We compare NLDMseq with other three popular alternatives, Cufflinks v2.2.0 [12], RSEM v1.2.9 [6] and MMSEQ v1.0.8 [4] for gene and isoform expression calculation. We use three real datasets and one simulated dataset for the validation of the performance of various methods. We also apply NLDMseq to find the DE genes and compare its performance with Cufflinks, RSEM and MMSEQ. Finally, we use an example dataset to compare the computation efficiency for the four approaches. The chart displaying the various study goals and the datasets used to accomplish each goal is shown in Table 1.

**Table 1:** Chart displaying the various study goals (row names) and the datasets (column names) used to accomplish each goal. Details of each dataset can be found in the section of Datasets

	MAQC	SELC	HBC	Simulation
Compare gene expression calculation	*√*	*√*		*√*
Compare isoform expression calculation			*√*	*√*
Compare DE detection performance	*√*			
Compare computation efficiency		*√*		

### Application to real data

We evaluate the proposed approach, NLDMseq, on the estimation of gene and isoform expression in terms of accuracy using three real datasets and considering both single-end and paired-end data.

#### Estimated isoform- and exon-specific read sequencing rate

**Fig. 3:**
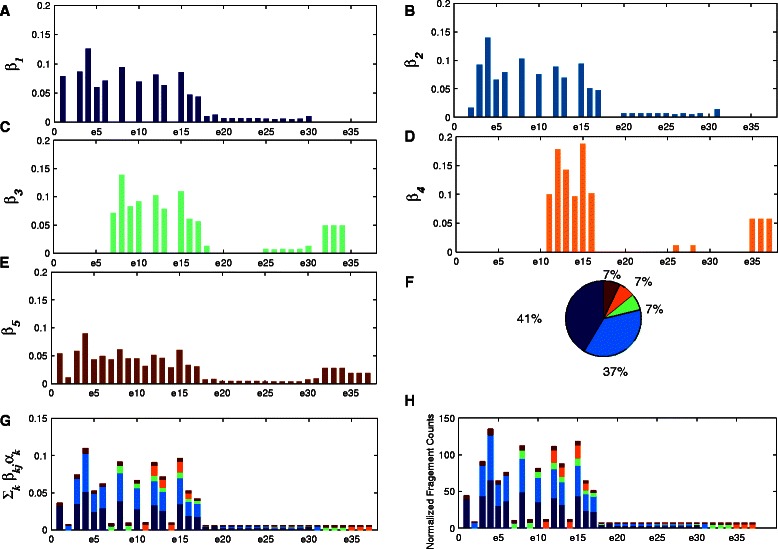
Consistency between isoform- and exon-specific read sequencing rates with observed read counts. The estimated isoform- and exon-specific read sequencing rates for the four isoforms of gene ENSG00000168394 with 37 exons (including 20 junction exons) are shown in **a** ∼**d**, and **e** is for the special noise isoform. The pie chart in **f** shows the fractions of the abundance from the five isoforms. The constitution of the summarized sequencing rate for each exon is shown in **g** with contributions from all the five isoforms. Subplot **h** shows the normalized fragment count for each exon with the contributions from the multiple isoforms

One advantage of NLDMseq is that it is able to model the isoform- and exon-specific read sequencing rate which is thought to be the main reason for the non-uniformity of read distribution in RNA-Seq data. Before evaluating the accuracy of expression calculation, we use a randomly selected example from the MAQC dataset as shown in Fig. 3 to demonstrate that the estimated isoform- and exon-specific read sequencing rate is consistent with the observed fragment counts. In this example, gene ENSG00000168394 contains 5 isoforms (including the one for handling noise) and 37 exons (including 20 junction exons). Here we show the results from HBR sample in the SRA010153 data. In our model, the read sequencing rates for exon *j* of isoform *k* is represented by *β*_*kj*_. The subplots a ∼e in Fig. 3 show the estimated exon-specific *β*_*kj*_ for each isoform. We can see that the sequencing rates for different exons of the same isoform are really different. From subplot f in Fig. 3 we can find the fraction of abundance for every isoform, *α*_*k*_. For each exon, we summarize the sequencing rates contributed from all isoforms, $\sum _{k}\beta _{\textit {kj}}\alpha _{k}$, for each exon as shown in subplot g in Fig.3. We can see that the constitution of the overall sequencing rate is diverse across exons. The subplot h in Fig.3 shows the normalized fragment count for each exon with the contributions from multiple isoforms represented by the same colors to those in Fig.3 g. By comparing the distribution of sequencing rates in Fig.3 g and the distribution of fragment counts in Fig.3 h, we can observe the identical variation pattern. This demonstrates that the estimated parameters of our model, *β*_*kj*_ and *α*_*k*_, are consistent with the observed data. In order to consider reads caused by noise or undiscovered isoforms, we include a special isoform which contains all exons of this gene. We observe that the fraction of this special isoform is as low as 9 %, which is not greater than the fraction of known isoforms in this example, showing that most sequenced fragments are useful and few are credited to noise and undiscovered isoforms.

#### Accuracy on gene expression estimation

We first use the well-studied MAQC dataset to justify the accuracy of NLDMseq on gene expression estimation. Each sample in this dataset contains multiple lanes. NLDMseq processes multi-lane data automatically and obtain the intrinsic fraction of multi-isoform abundance based on multi-lane information. We also apply Cufflinks, RSEM and MMSEQ to data coming from each individual lane and calculate the average gene expression for each sample. The Pearson correlation coefficients of gene expression estimation with the qRT-PCR measurements for the 740 qRT-PCR validated genes in the three samples are calculated as shown in Table 2. The higher the numbers, the better the performance of the methods. The related scatter plots can be found in Additional file 1: Figure S1. We can see that NLDMseq obtains the most consistent expression measurements compared with qRT-PCR results for all the three comparison cases. Additional file 2: Figure S2, Additional file 3: Figure S3, Additional file 4: Figure S4 show the consistency of estimated gene expression from various methods. We find methods obtain fair consistent results generally except Cufflinks. The discrepancy between results from different approaches is mainly located for lowly expressed genes, showing the difficulty in estimating low expression.

**Table 2:** The Pearson correlation coefficients of estimated expression with qRT-PCR measurements using MAQC dataset

Dataset	Cufflinks	RSEM	MMSEQ	NLDMseq
SRA010153 (HBR)	0.8033	0.8117	0.8000	0.8442
SRA010153 (UHR)	0.8238	0.8265	0.8356	0.8585
SRA012427 (UHR)	0.8107	0.8345	0.8430	0.8481

Samples HBR and UHR in SRA010153 data and sample UHR in SRA012427 data are used

We next use the real SELC data with qRT-PCR validation to further verify the performance of NLDMseq on gene expression calculation. Table 3 shows the calculated log-ratio of each qRT-PCR validated gene in one of the two comparisons, S vs. NS and C vs. NC. We compare the consistency of results from various methods with qRT-PCR measurements, and calculate the absolute error rate (AER) as shown in the bracket after each calculated log-ratio. The lower the AER values, the better the performance of the methods. We find that all the four methods produce inconsistent direction of fold change between RNA-seq and qRT-PCR for gene SCGB1A1. The three approaches, Cufflinks, RSEM and NLDMseq, obtain relatively low AER, while MMSEQ obtains higher AER.

**Table 3:** Comparison results of various methods for SELC dataset on calculated log _2_ fold change of gene expression

Comparison	Gene	qRT-PCR	Cufflinks	RSEM	MMSEQ	NLDMseq
S vs. NS	S100A8	+1.15	+1.13(0.02)	+2.72(1.38)	+3.20(1.78)	+1.65(0.44)
	S100A9	+0.83	+1.69(1.03)	+1.79(1.16)	+1.86(1.24)	+1.75(1.10)
	CYP4F2	+1.27	+4.12(2.24)	+3.59(1.82)	+9.51(6.49)	+2.09(0.64)
C vs. NC	CCL20	+4.01	+6.00(0.50)	+4.66(0.16)	+5.32(0.32)	+7.08(0.76)
	IL8	+1.50	+1.51(0.01)	+1.21(0.19)	+1.67(0.11)	+1.05(0.30)
	SCGB3A1	+0.51	+1.65(2.23)	+1.76(2.45)	+1.85(2.63)	+1.82(2.57)
	SCGB1A1	−0.48	+1.37(3.89)	+1.56(4.26)	+1.02(3.13)	+1.62(4.39)
Average AER		NA	1.42	1.63	2.24	1.46

The symbol “ +” stands for up-regulation and “ −” for down-regulation. Numbers in the brackets stand for absolute error rates (AER) compared with qRT-PCR results. AER is calculated by |(*r*−*e*)/*r*|, where |·| stands for absolute, and *r* and *e* represent qRT-PCR and RNA-seq measurements, respectively. The last line shows the average AER for each method

#### Accuracy on isoform expression estimation

We use the HBC dataset to justify the isoform expression obtained from NLDMseq and compare it with Cufflinks, RSEM and MMSEQ. We used the UCSC knownGene transcriptome annotation (NCBI36/hg18) for obtaining all the annotation information for the eight qRT-PCR validated isoforms related to genes TRAP1, ZNF581, WISP2 and HIST1H2BD. We apply NLDMseq, Cufflinks, RSEM and MMSEQ to this dataset and calculate the expression of the eight qRT-PCR validated isoforms. The log-ratios obtained from different methods for the 16 regulations of the eight qRT-PCR validated isoforms are shown in Table 4. We consider eight regulations of the eight isoforms under two conditions and eight regulations of the two isoforms belonging to the same gene under the same condition. Details of each comparison can be found in Additional file 7: Table S3. It can be seen that NLDMseq obtains the most consistent results compared with qRT-PCR for all the 16 comparison cases, while Cufflinks, RSEM and MMSEQ all produce 4 inconsistent results. NLDMseq also produces the lowest AER among all the approaches. This comparison shows the superiority of NLDMseq on the calculation of isoform expression.

**Table 4:** Comparison results of various methods for HBC dataset on calculated log _2_ fold change of isoform expression

Comparison	qRT-PCR	Cufflinks	RSEM	MMSEQ	NLDMseq
Case 1	−0.4	−0.81(1.03)	−0.41(0.02)	−0.74(0.85)	−0.91(1.28)
Case 2	−0.5	−0.73(0.46)	−0.83(0.66)	−0.57(0.14)	−0.59(0.18)
Case 3	−0.9	+4.52(6.02)	+4.91(6.46)	+4.42(6.02)	−0.34(0.62)
Case 4	−1.0	+3.36(4.36)	+4.44(5.44)	+4.59(5.59)	−0.04(0.96)
Case 5	−0.3	−1.74(4.80)	−0.93(2.10)	−1.29(3.30)	−2.41(7.03)
Case 6	−1.0	−1.11(0.11)	−0.71(0.29)	−1.08(0.08)	−1.29(0.29)
Case 7	+1.2	+1.12(0.07)	+1.34(0.12)	+1.47(0.23)	+0.37(0.69)
Case 8	+1.0	+1.26(0.26)	+1.56(0.57)	+1.67(0.67)	+1.37(0.37)
Case 9	−5.6	−5.70(0.02)	−5.40(0.04)	−5.50(0.02)	−7.76(0.39)
Case 10	−4.5	−4.67(0.04)	−4.71(0.05)	−4.83(0.07)	−5.61(0.25)
Case 11	+0.4	+0.52(0.30)	0.01(0.98)	+0.34(0.15)	+0.26(0.35)
Case 12	+1.5	+1.71(0.14)	+0.81(0.46)	+1.00(0.33)	+2.38(0.59)
Case 13	−4.7	−2.74(0.42)	−2.92(0.38)	−4.28(0.09)	−1.07(0.77)
Case 14	−5.2	−5.89(0.13)	−4.51(0.13)	−4.70(0.10)	−4.26(0.18)
Case 15	−5.4	+3.57(1.66)	+1.83(1.34)	+1.79(1.33)	−0.21(0.96)
Case 16	−5.9	+1.11(1.19)	+0.21(1.04)	+1.37(1.23)	−3.33(0.44)
No. of inconsistency	NA	4(1.31)	4(1.25)	4(1.26)	0(0.96)

The symbol “ +” stands for up-regulation and “ −” for down-regulation. Numbers in the brackets stand for absolute error rates (AER) compared with qRT-PCR results. AER is calculated by |(*r*−*e*)/*r*|, where |·| stands for absolute, and *r* and *e* represent qRT-PCR and RNA-seq measurements, respectively. The last line shows the number of inconsistent regulation and the average AER for each method

**Fig. 4:**
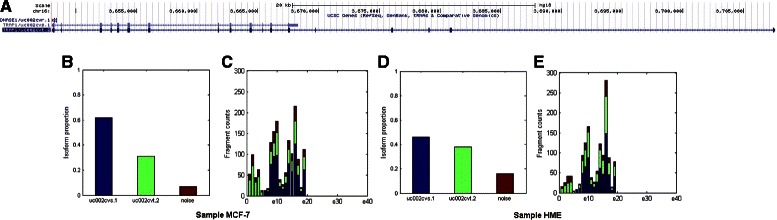
Isoform expression estimation of gene TRAP1 under cell lines HME and MCF-7. Subplot **a** displays the gene mode from Ensembl. Subplots **b** and **c** present calculation results from NLDMseq for cell line HME, and **d** and **e** are for cell line MCF-7. Histograms in **b** and **d** are the expectation of fraction of isform abundance for the two cell lines. Histograms in **c** and **e** show the observed fragment counts mapped to each exon (including junction exon). We use different colors to represent the contributions from multiple isoforms

**Fig. 5:**
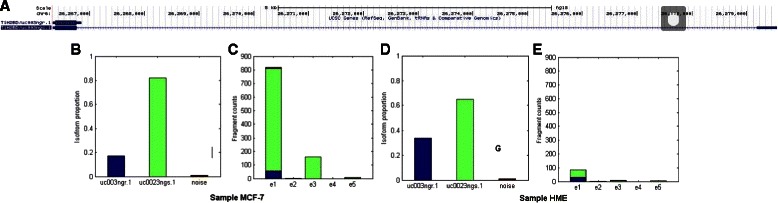
Isoform expression estimation of gene HIST1H2BD under cell lines HME and MCF-7. Subplot **a** displays the gene mode from Ensembl. Subplots **b** and **c** present calculation results from NLDMseq for cell line HME, and **d** and **e** are for cell line MCF-7. Histograms in **b** and **d** are the expectation of fraction of isform abundance for the two cell lines. Histograms in **c** and **e** show the observed fragment counts mapped to each exon (including junction exon). We use different colors to represent the contributions from multiple isoforms

We find the regulations between isoforms of genes TRAP1 and HIST1H2BD under cell lines HME and MCF-7 (rows 3,4,15 and 16 in Table 4) are incorrectly calculated by Cufflinks, RSEM and MMSEQ. In contrast, NLDMseq obtains consistent results for the two genes with qRT-PCR measurements. This shows the difficulties in partitioning the mapping fragments into contributions from the multiple isoforms. Figures 4 and 5 show the isoform expression calculations for genes TRAP1 and HIST1H1BD, respectively. The expectation of the isoform percentage is obtained as shown in subplots b and d in Figs. 4 and 5. We can see that for gene TRAP1 the percentage of noise is 16 % and 7 % for cell lines HME and MCF-7, respectively. For both cell lines, the percentage of isoform uc002cvs.1 is higher than that of isoform uc002cvt.2. We partition the mapping reads of each exon according to the obtained isoform percentage as shown in subplots c and e in Figs. 4 and 5. After normalization on the isoform length, the obtained expression of uc002cvt.2 is lower than that of uc002cvs.1 which is consistent with qRT-PCR. Similarly, we can see from Fig. 5 that the expected percentage of noise for gene HIST1H1BD is as low as 1 % and the percentage of isoform uc003ngr.1 is lower than that of isoform uc003ngs.1. After separating the mapping fragments, the normalized isoform expression of uc003ngr.1 is thus lower than that of uc003ngs.1 showing consistency with qRT-PCR results.

### Simulation study

**Fig. 6:**
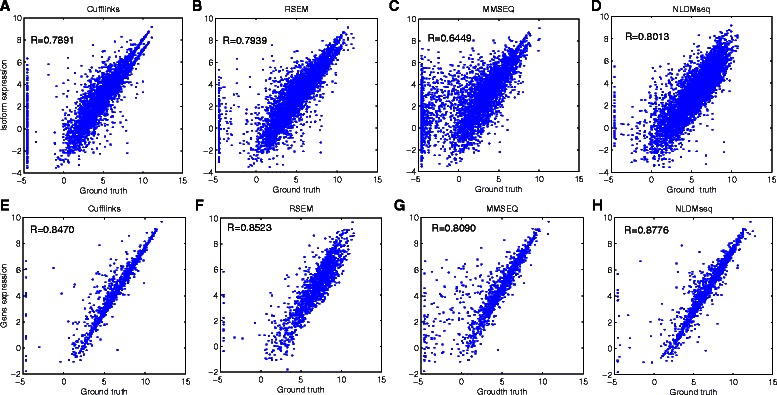
Comparison of expression estimation accuracy for simulated single-end data. Scatter plots of the expression estimates versus the true expression values for the simulated single-end data are showed as well as the Pearson correlation coefficients. The upper panels **a** ∼**d** shows results for isoform expression and the lower panels **e** ∼**h** for gene expression. NLDMseq is compared with Cufflinks, RSEM and MMSEQ

**Fig. 7:**
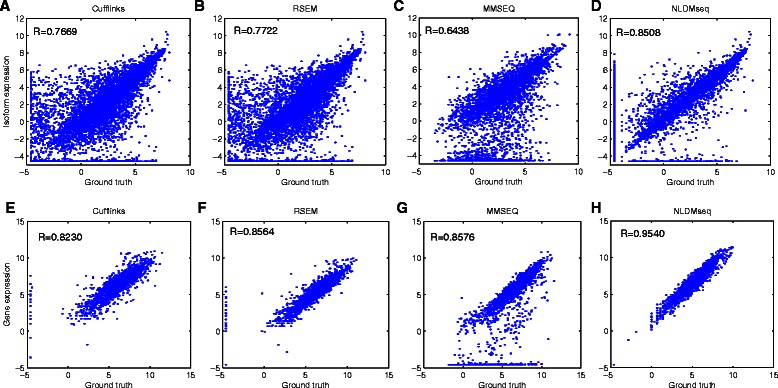
Comparison of expression estimation accuracy for simulated paired-end data. Scatter plots of the expression estimates versus the true expression values for the simulated paired-end data are showed as well as the Pearson correlation coefficients. The upper panels **a** ∼**d** shows results for isoform expression and the lower panels **e** ∼**h** for gene expression. NLDMseq is compared with Cufflinks, RSEM and MMSEQ

Considering that the real abundance for a large number of transcripts can not be available in reality, we use the generated simulation data to validate the consistency of NLDMseq under the data simulated with it in terms of accuracy in gene and isoform expression calculation. Figures 6 and 7 show the scatter plots and Pearson correlation coefficients of the gene and isoform expression estimation with the true expression values for the single-end and paired-end simulated data, respectively. We can see from these figures that the obtained correlations for isoform expression are significantly lower than those for gene expression, showing that estimation of isoform expression is more difficult than that of gene expression. It is not surprising that NLDMseq obtains the highest consistency with the ground truth for both data since the data is generated using our model and is therefore biased to it. In spite of this, the other three methods also present reasonable results. Cufflinks and RSEM obtain the similar accuracy to NLDMseq for single-end data, while NLDMseq shows outstanding superiority for paired-end data. We also notice that the performance of RSEM, MMSEQ and NLDMseq gets improved when paired-end data is used for gene expression calculation while that of Cufflinks does not. In addition, all the four approaches obtain more noise in the lower end of calculated isoform expression for paired-end data indicating that more concerns should be raised for data involved more junction fragments when isoform expression is of interest.

### Application to DE analysis

The calculated gene and isoform expression from NLDMseq can be used in the downstream analysis of RNA-seq, such as DE gene and isoform detection. We apply NLDMseq to MAQC dataset to show the usefulness of our method in DE analysis and compare it with the other alternatives. The 279 qRT-PCR validated DE genes with high certainty are taken as the golden standard to verify DE analysis using our method. To reach this goal, we combine NLDMseq with PPLR [25] approach, which is a hierarchical Bayesian model previously applied to microarray analysis and has been implemented in the R package *puma* [26]. PPLR combines the biologically replicated expression measurements and considers measurement uncertainty associated with these estimates. This approach can be applied to the expression estimated from both microarray and RNA-seq. As we introduced in the section of ‘Methods’, NLDMseq is able to produce a level of measurement uncertainty associated with expression estimate. We propagate this measurement uncertainty into PPLR to perform DE analysis. Details of the usage of PPLR can be found in the documentation of *puma*. Cufflinks, RSEM and MMSEQ are combined with the embedded DE analysis approaches, Cuffdiff [27], EBSeq [28], and MMDiff [29], respectively.

**Fig. 8:**
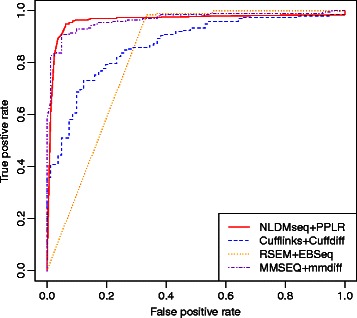
ROC curves of DE analysis for selected RCR-validated multi-isoform genes in MAQC dataset. NLDMseq is combined with PPLR to propagate measurement uncertainty in DE analysis. Cufflinks, RSEM and MMSEQ are combined with embedded DE analysis methods, Cuffdiff, EBSeq and mmdiff, respectively. The AUCs for NLDMseq, Cufflinks, RSEM and MMSEQ are 0.9659, 0.8793, 0.8275 and 0.9625, respectively

We use the receiver operating characteristic (ROC) curves to show the performance of various DE approaches. The higher the curve, the better performance of the examined method. Accordingly, a higher value of area under the ROC curve (AUC) indicates higher accuracy of the method. We plot the ROC curves for different DE approaches as shown in Fig. 8. We can see that NLDMseq combined with PPLR produces the competitive ROC curve compared to MMSEQ. NLDMseq and MMSEQ give the values of AUC of 0.9625 and 0.9659, respectively. In contrast, Cufflinks and RSEM present lower ROC curves and result in AUCs of 0.8793 and 0.8275, respectively. This example shows the competitive performance of our method in DE analysis.

### Comparison on computation time

Finally, we use the data of NC phenotype in the SELC data to compare the computation efficiency of various approaches. This data contain 22 million paired-end reads. The expression computation time of the four methods are shown in Table 5. Since all methods use similar read alignment software, we exclude the running time for read alignment and compare only the running time for expression computation. Using parallel computing with four threads for all methods, Cufflinks is the most time-consuming for processing this data. The running time of MMSEQ is close to that of RSEM. NLDMseq obtains the least running time showing the superior computation efficiency among the four methods.

**Table 5:** The expression computation time (in minutes) of various methods for the NC phenotype of the SELC dataset. All methods use parallel computing involving four threads. We show the running time for expression computation and exclude the time for read alignment. Computation time is obtained on a 3.2 GHz quad-core Intel machine with 16G RAM

Method	Cufflinks	RSEM	MMSEQ	NLDMseq
Computation time	70	44	50	26

## Conclusion

In this contribution, we have presented a latent variable model, NLDMseq, to accurately estimate gene and isoform expression from RNA-Seq data given a known annotation. NLDMseq handles the two major difficulties in expression calculation in RNA-Seq data analysis, read mapping ambiguity and non-uniformity of read distribution, by adopting a latent model to discover the hidden pattern of read sequencing and modeling the isoform- and exon-specific read sequencing rates. Unlike many latent variable models which simulate the sequencing of each individual read, NLDMseq models normalized read counts for each exon without dealing with nucleotide constitution of each read. This significantly reduces the computation load of the model. We have used the replicate information of multiple lanes for a single library run to achieve read sequencing rate for each isoform-specific exon which shows the particular sequencing bias for this exon. We have constructed junction-exons to deal with reads mapped to the region of exon junction. For accommodation of noise reads and reads mapped to undiscovered isoforms, we have considered a special isoform which contains all the exons of a gene. We have found that the fraction of this special isoform is usually lower than that of known isoforms indicating that most reads are useful in calculation of expression for known isoforms and there are few reads which are due to noise and undiscovered isoforms.

We have employed simulation and qRT-PCR validated real data to verify the performance of our method in terms of accuracy in the calculation of gene and isoform expression. Results have shown that NLDMseq is comparable to and in some cases outperforms the other competing methods. We have also applied NLDMseq to DE analysis by combining a previously designed DE analysis method, PPLR, which has been successfully applied to microarray analysis. This DE analysis approach was tested on a well-studied qRT-PCR validated benchmark. Results have shown that our approach have presented competitive performance compared with other popular methods. We also used a dataset to examine the running time of our approach. The comparison results indicate that our approach is computationally efficient compared with the other alternatives.

One advantage of our method is that isoform- and exon-specific sequencing rate can be estimated to account for different sequencing bias for each isoform-specific exon. However, we find the distribution of the observed read counts is over-dispersed compared with the obtained sequencing rates as shown in subplots g and h in Fig. 3. To address this problem, a Dirichlet prior can be put over the sequencing rates for isoform-specific exons in the future work. We expect this Dirichlet-Multinomial model of observed counts would further improve the performance of our model.

We have used an example of detecting DE genes to show the application of NLDMseq to DE analysis. As for the detection of differential alternative splicing, NLDMseq can also be combined with available DE analysis method, such as PPLR, to find DE isoforms by using the isoform expression calculated in Eq. (7). Moreover, by using the posterior distribution of the percentage of isoforms, {*θ*_*l*_}, obtained from the variational EM estimation, it is also possible to detect differential isoform usage, where multiple isoforms of a single gene are expressed, but at different proportions between two groups of samples. This also provides a possibility of identifying alternative splicing.

## Additional files

Additional file 1
**Figure S1.** Comparison of expression estimation accuracy at gene level for the MAQC data. Pearson correlation coefficients of the expression estimates for the 740 qRT-PCR validated multi-isoform genes with the qRT-PCR results are shown. The upper panel shows results for the single-end sample HBR, the middle panel for the single-end sample UHR and the lower panel for the paired-end sample UHR. NLDMseq (4th column) is compared with Cufflinks (1st column), RSEM (2nd column) and MMSEQ (3rd column). (EPS 222 kb)

Additional file 2
**Figure S2.** Consistency of estimated gene expression from various methods for the single-end sample HBR in the MAQC data. Scatter plots of the estimated expression of the the 740 qRT-PCR validated genes from various methods for the single-end sample HBR in the MAQC data are shown in the lower-left part of the figure. Pearson correlation coefficients of the expression estimates between approaches are shown in the upper-right part of the figure. (EPS 114 kb)

Additional file 3
**Figure S3.** Consistency of estimated gene expression from various methods for the single-end sample UHR in the MAQC data. Scatter plots of the estimated expression of the the 740 qRT-PCR validated genes from various methods for the single-end sample UHR in the MAQC data are shown in the lower-left part of the figure. Pearson correlation coefficients of the expression estimates between approaches are shown in the upper-right part of the figure. (EPS 114 kb)

Additional file 4
**Figure S4.** Consistency of estimated gene expression from various methods for the paired-end sample UHR in the MAQC data. Scatter plots of the estimated expression of the the 740 qRT-PCR validated genes from various methods for the paired-end sample UHR in the MAQC data are shown in the lower-left part of the figure. Pearson correlation coefficients of the expression estimates between approaches are shown in the upper-right part of the figure. (EPS 114 kb)

Additional file 5
**Table S1.** Name list of the 740 genes used for gene expression validation in MAQC data. This table shows the name list of the 740 qRT-PCR validated genes which are used to validate gene expression calculation in MAQC data. (XLS 243 kb)

Additional file 6
**Table S2.** Name list of the 279 genes used for comparison on DE analysis in MAQC data. This table shows the name list of the 279 qRT-PCR validated DE genes with high certainty, which are used to validate DE analysis approaches in MAQC data. (XLS 39.5 kb)

Additional file 7
**Table S3.** Description of 16 comparison cases for isoform expression validation in HBC data. This table shows the 16 comparison cases in HBC dataset. Each gene contains four comparison cases, among which two are from the same transcript with comparison between the two cell lines, and two from the same cell line with comparison between the two transcripts. (XLS 18.5 kb)
